# Plasma Levels of Neudesin and Glucose Metabolism in Obese and Overweight Children

**DOI:** 10.3389/fendo.2022.881524

**Published:** 2022-07-14

**Authors:** Edoardo Vergani, Carmine Bruno, Clelia Cipolla, Diego Currò, Antonio Mancini

**Affiliations:** ^1^ Dipartimento di Medicina e Chirurgia Traslazionale, Fondazione Policlinico Universitario A. Gemelli IRCCS - Università Cattolica del Sacro Cuore, Roma, Italy; ^2^ Dipartimento di Scienza e Salute della donna e del bambino, Fondazione Policlinico Universitario A. Gemelli IRCCS - Università Cattolica del Sacro Cuore, Roma, Italy; ^3^ Dipartimento di Sicurezza e Bioetica, Sezione di Farmacologia, Università Cattolica del Sacro Cuore - Fondazione Policlinico Universitario A. Gemelli IRCCS, Roma, Italy

**Keywords:** neudesin, energy homeostasis, childhood obesity, insulin-resistance, biomarkers

## Abstract

Childhood overweight and obesity are among the major health problems of modern times, especially in Western countries, due to their association with increased cardiovascular and cancer risk in adulthood. Neudesin, a recently discovered peptide secreted mainly in the brain and adipose tissue, is being investigated for its possible activity as a negative regulator of energy expenditure. We conducted a cross-sectional observational preliminary study with the aim of testing the hypothesis that plasma levels of neudesin can be modified in obese and overweight children and to evaluate any possible relationship between plasma neudesin levels and metabolic and anthropometric parameters. 34 Children (Tanner’s stage 1) were included and divided in two groups according to Cole’s criteria. Group A included obese and overweight children (23 patients, 17 females and 6 males, aged 4-10 years); Group B included healthy normal-weight children (11 subjects, 7 females and 4 males, aged 3-10 years). Metabolic (glucose and insulin, total, LDL- and HDL-cholesterol, triglycerides, uric acid) and hormonal (fT3, fT4, TSH, IGF-1, leptin) parameters were evaluated. HOMA-IR and QUICKI index and the area under the curve (AUC) of glucose and insulin after oral glucose load were calculated in obese and overweight children. Neudesin was measured by ELISA. Neudesin levels were significantly higher in obese/overweight children than in controls. In obese and overweight children, plasma neudesin levels were significantly directly correlated with blood glucose and glucose AUC. Taken together, these results, although preliminary, may suggest a possible age-related role of neudesin in glucose homeostasis in obese/overweight children.

## Introduction

Childhood overweight and obesity are among the major health problems of modern times, especially in Western countries, since being overweight early in life can lead to some complications (e.g., retarded growth and behavioral disturbances). Even worse consequences, such as cardiovascular diseases and cancer, are described in adulthood (1).

Overweight, obesity and metabolic syndrome (MetS) are closely related to each other. In adulthood, hypertriglyceridemia, hyperglycemia, hypertension, and increased waist circumference, which in turn is related to insulin resistance (IR), are some of the criteria used to define the MetS. However, criteria for establishing the diagnosis of MetS in childhood have not yet been clearly defined (2, 3), although both fatty liver and increased intima thickness have been described already in overweight children (4, 5). The need to shed light on the MetS in childhood has led to the identification of some peptides whose serum levels are closely related to the development of IR (2, 6–8), including neudesin.

Neudesin, also known as neuron-derived neurotrophic factor (NENF), is a secreted protein of 172 amino acids, widely expressed in the brain and adipose tissue, but also detectable in the heart, kidneys, and lungs (9). As a member of the membrane-associated progesterone receptor (MAPR) protein family, neudesin presents a cytochrome b5-like heme/steroid binding domain, which is crucial for initiating intracellular signalling pathways (9, 10). Neudesin activates the mitogen-activated protein kinase (MAPK) and phosphatidylinositol-3 kinase (PI3K) pathways through G_i_/G_o_ proteins in neurons (11–13). In neural precursor cells, neudesin increases cAMP levels and induces cell differentiation and transient proliferation through the protein kinase A (PKA) pathway and other signalling pathways, suggesting that it binds to G_s_ protein-coupled receptors in these cells (10). In addition to inducing proliferation and differentiation of neural precursor cells and having neurotrophic activity (14), neudesin also promotes, through the activation of the before mentioned signaling pathways, the invasiveness and tumorigenicity of cancer cells (13, 15) in experimental models.

In neudesin gene knockout mice, increased energy expenditure, lipolysis in white adipose tissue, heat production of brown adipose tissue, and a protection to IR related to high-fat diet and decreased food intake were shown (16); these data have thus led to focus attention on the action of neudesin as a possible negative regulator of energy expenditure. However, few and discordant studies are available in the literature on the levels of neudesin in biological fluids of patients (17–23) and no data on childhood obesity are yet available.

The aim of our study was to gain insight whether neudesin may play a role in childhood obesity. To implement this aim, we analyzed through an observational cross-sectional study whether the plasma levels of neudesin in overweight and obese children were significantly different from those detectable in normal weight children. Furthermore, we verified whether there was any relationship between plasma levels of neudesin and metabolic and anthropometric parameters.

## Methods

### Population Enrollment

A total of 34 children (10 males and 24 females) of Caucasian origin, in prepubertal age (3-10 years, Tanner stage I) (24), admitted to the “A. Gemelli” University Hospital, were enrolled in the study. Informed consent was obtained from the children’s parents and the study protocol was approved by our Institutional Review Board. Since BMI values in childhood, unlike adulthood, depend on age and sex, the standard deviation (SD) of BMI was used for the analysis of the data, considering as overweight children those with BMI greater than 1.6 SD (corresponding to 85th centile) and obese children those with a BMI greater than 1.8 SD (corresponding to 95th percentile). Taking as a reference the work of Cole et al. (2000) (25), two groups were defined based on BMI: group A represented obese/overweight children (23 patients, 17 females and 6 males, aged 4-10 years) and group B included normal weight children (11 patients, 7 females and 4 males, aged 3-10 years). In group A, 17 children were obese and 6 were overweight according to Cole’s criteria.

Based on phenotypic, anamnestic, and hormonal data, the following conditions were excluded: monogenic obesity; syndromic obesity; obesity due to endocrine diseases (Cushing syndrome, hypothyroidism, hypopituitarism); obesity due to diencephalic or encephalic lesions or malformation; iatrogenic obesity related to drugs taken during the previous 6 months (antiepileptics, antidepressant, corticosteroids); acute or chronic inflammatory diseases; type 2 diabetes mellitus (T2DM) and type 1 diabetes mellitus (T1DM).

### Anthropometric Parameters

For each child the following parameters were measured: standing height (Harpenden stadiometer), naked weight (precision scales), waist circumference (WC; flexible meter). Italian growth charts have been used as reference standards for height and weight. Waist circumferences have been compared to the values set out in McCarthy et al. (2001) (26). Body mass index (BMI) was calculated by the formula: weight (kg)/height² (meter).

### Biological Sample Collection

All children underwent venous sampling at 08.00 am, after an overnight fasting, collecting blood samples by two pyrogen-free tubes, one containing lithium-heparin as anticoagulant and one without anticoagulant to obtain serum. All samples were centrifuged immediately after basal sampling at a temperature of 4°C at 2700 rpm for 10 minutes. Plasma or serum aliquots were then separated and stored at a temperature of -80°C. We determined plasmatic glucose, total cholesterol, HDL-cholesterol, triglycerides, uric acid, leptin, and neudesin; and serum insulin, insulin-like growth factor-1 (IGF-1), free triiodothyronine (fT3), free thyroxine (fT4), and thyroid stimulating hormone (TSH). Moreover, in the population of obese and overweight children, an oral glucose tolerance test (OGTT) was performed (oral glucose dose of 1.75 g/kg of ideal weight, max 75 g), with blood samples taken every 30 min until 120 minutes to evaluate glucose and insulin response. QUICKI index was calculated according to the formula 1/log [fasting insulin (μUI/ml)] + log [fasting glucose (mg/dl)] (27). Homeostasis model of insulin resistance (HOMA-IR), an insulin resistance index, was calculated according to the formula: [fasting insulin (μUI/ml)] * [fasting glucose (mg/dl)]/405 (28). In the absence of a unanimous consensus about the definition of insulin resistance in pediatric age, we chose a cut-off of HOMA greater than 2.67 in males and 2.22 in females according to the results obtained by Kurtoglu et al. (2010) in a population of 268 obese children and adolescents (29) and according to our previous study (30).

Plasma concentrations of glucose, total cholesterol, HDL-cholesterol, triglycerides, uric acid were measured by using enzymatic assays. The intra-and inter-assay coefficients of variation (CV) for total cholesterol, triglycerides, and uric acid were < 1.5% and < 2.5%, respectively. The intra-and inter-assay CV for HDL-cholesterol were < 2.5%, and < 3.0%, respectively. LDL cholesterol was calculated by the Friedewald’s equation: LDL = total cholesterol - (HDL + triglycerides/5). Serum concentrations of insulin, IGF-1, TSH, fT3, and fT4 were measured by using immunochemiluminometric assays on a Roche Modular E170 analyzer (Roche Diagnostics, Indianapolis, IN, USA). The intra- and inter-assay CV for insulin, IGF-1, TSH, fT3, and fT4 were < 5.0% and < 7.0%, respectively.

Plasma levels of leptin were measured using an enzyme-linked immunosorbent assay (Elisa) kit (DRG Instruments GmbH, Marburg, Germany) according to the manufacturer’s protocol. The detection limit of leptin in this assay is 1.0 ng/ml. The intra- and inter-assay CV are < 5.9% and < 8.6%, respectively. Plasma concentrations of neudesin were measured by an Elisa kit (RayBiotech, Norcross, GA, USA; Cat. N. ELH-NEUD) following the protocol provided by the manufacturer. The Elisa kit limit of detection of human neudesin is 14.5 pg/ml and the calculated overall intra-assay and inter-assay CV are < 10% and < 12%, respectively.

### Statistical Analysis

The statistical analysis was performed using GraphPad Prism 8. A formal sample calculation is not necessary since the present study is considered a pilot. However, according to the rules proposed by Birkett and Day for internal pilot studies (31), the sample size is estimated at 20 patients. D’Agostino and Pearson test was performed preliminarily to evaluate the distribution of data in the population studied. Continuous variables are expressed as mean ± SD. If a normal distribution of data was displayed, the results were analyzed by means of Student’s unpaired t-test to evaluate the differences between groups and Pearson coefficient for correlation analysis. On the other hand, if data did not show a normal distribution, Mann-Whitney test was performed to study the differences between groups and Spearman coefficient for correlation analysis. Multivariate regression analysis has been performed to confirm data regarding correlations, using neudesin as dependent variable and BMI, glucose and HOMA-IR as independent variables. The level of significance has been set at 0.05.

## Results

BMI of obese/overweight children was significantly higher than that of normal-weight children (24.04 ± 2.51 kg/m^2^ vs 15.6 ± 2.05 kg/m^2^), by definition. The wrist, arm, waist, and hip circumferences in obese/overweight children were 15.17 ± 1.19 cm, 25.64 ± 2.55 cm, 72.85 ± 6.87 cm, and 83.69 ± 8.99 cm, respectively. The waist to hip circumference ratio was 0.88 ± 0.09.

**Figure 1:**
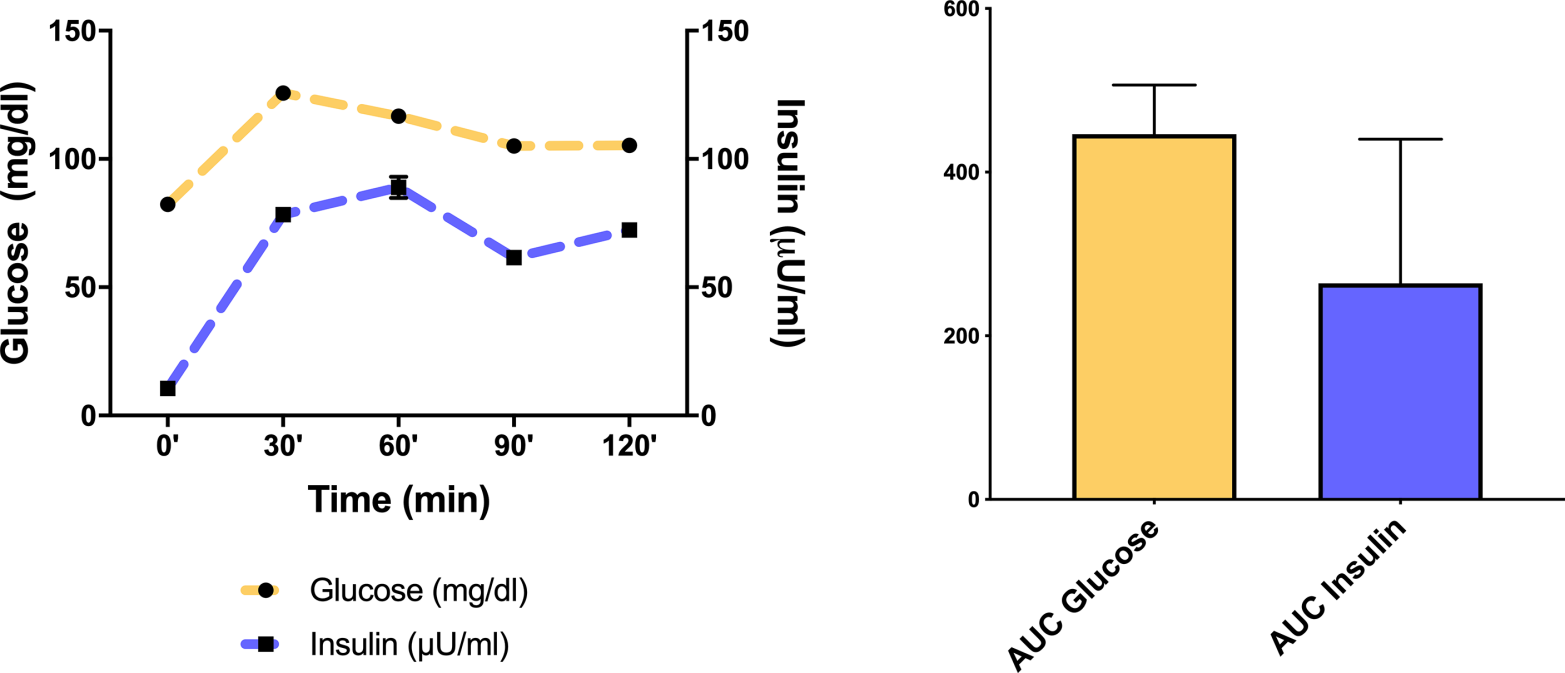
Glucose and insulin curve at 0’, 30’, 60’, 120’ minutes after oral glucose load and mean ± SD area under the curve (AUC) of glucose and insulin in the obese/overweight children group.

**Figure 1** shows glucose and insulin curve at 0’, 30’, 60’, 120’ minutes after oral glucose load and area under the curve (AUC) of glucose and insulin in obese/overweight children group.

**Table 1** shows the general, metabolic, and hormonal parameters in the two groups studied. As expected, obese and overweight children had significantly higher blood glucose, total and LDL cholesterol, triglycerides, and leptin levels than those with normal weight. Moreover, as an evidence of insulin-resistance state, group A showed higher HOMA-IR and lower QUICKI than healthy controls.

**Table 1:** Mean ± SD of general, metabolic, hormonal characteristics and neudesin levels in the two groups.

	GROUP A (17 Females, 6 Males)	GROUP B (7 Females, 4 Males)
**Age (months)**	99.20 ± 19.75	84.30 ± 26.17
**BMI (kg/m^2^)**	24.04 ± 2.51*	15.60 ± 2.05
**Blood Glucose (mg/dl)**	82.32 ± 6.64*	69.30 ± 2.34
**HOMA-ir**	2.24 ± 0.86*	0.68 ± 0.40
**QUICKI index**	0.34 ± 0.02*	0.42 ± 0.03
**Total Cholesterol (mg/dl)**	158.95 ± 23.31*	120.60 ± 19.09
**Triglycerides (mg/dl)**	77.95 ± 9.59*	46.60 ± 15.11
**HDL – Cholesterol (mg/dl)**	55.05 ± 11.70	51.20 ± 12.83
**LDL – Cholesterol (mg/dl)**	88.52 ± 22.50*	60.20 ± 4.27
**TSH (µU/ml)**	2.19 ± 0.75	2.47 ± 1.11
**FT4 (pg/ml)**	11.65 ± 1.37	11.05 ± 0.59
**FT3 (pg/ml)**	3.96 ± 0.40	4.09 ± 0.56
**IGF-1 (ng/ml)**	205.27 ± 65.06	155.67 ± 55.22
**Leptin (ng/ml)**	11.46 ± 6.73*	3.70 ± 1.98
**Neudesin (ng/ml)**	3.00 ± 1.48*	1.98 ± 0.05

Group A: Obese and Overweight children; Group B: normal-weight children. *p < 0.05.

**Figure 2:**
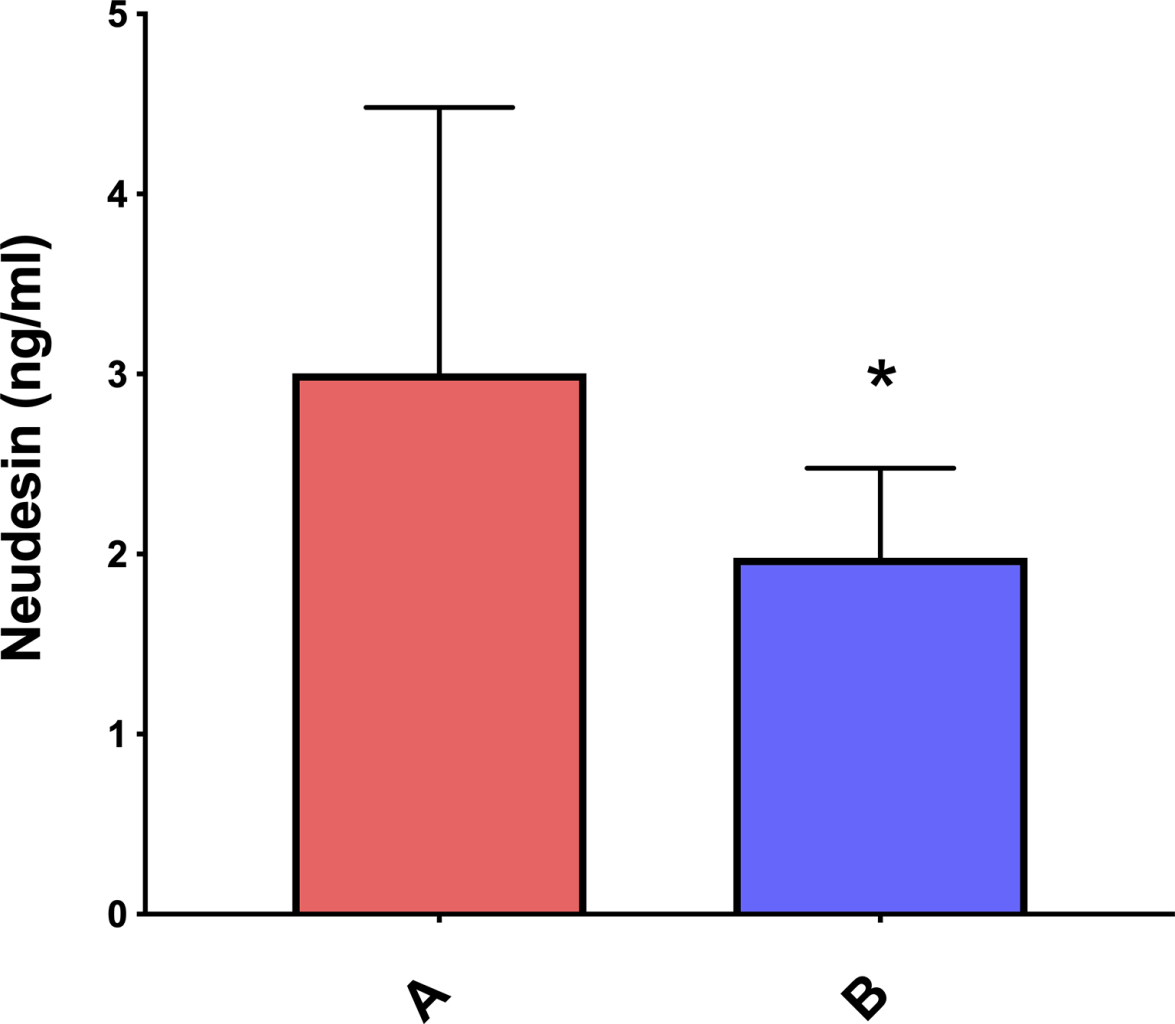
Mean ± SD neudesin plasmatic concentrations in obese/overweight versus normal-weight children. *p < 0.05. A= obese/pverweight children; B= normal-weight children.

Plasma neudesin levels were significantly higher in obese/overweight children than in controls (3.00 ± 1.48 ng/ml vs 1.98 ± 0.50 ng/ml, p = 0.03, **Figure 2**). Neudesin levels of the 17 obese children (3.04 ± 1.31 ng/ml) were significantly (p = 0.03) higher than those of the normal-weight children; moreover, the difference between neudesin levels of the 6 overweight children (2.91 ± 0.40 ng/ml) and those of the normal-weight children reached significance (p = 0.04) despite the low number.

**Figure 3:**
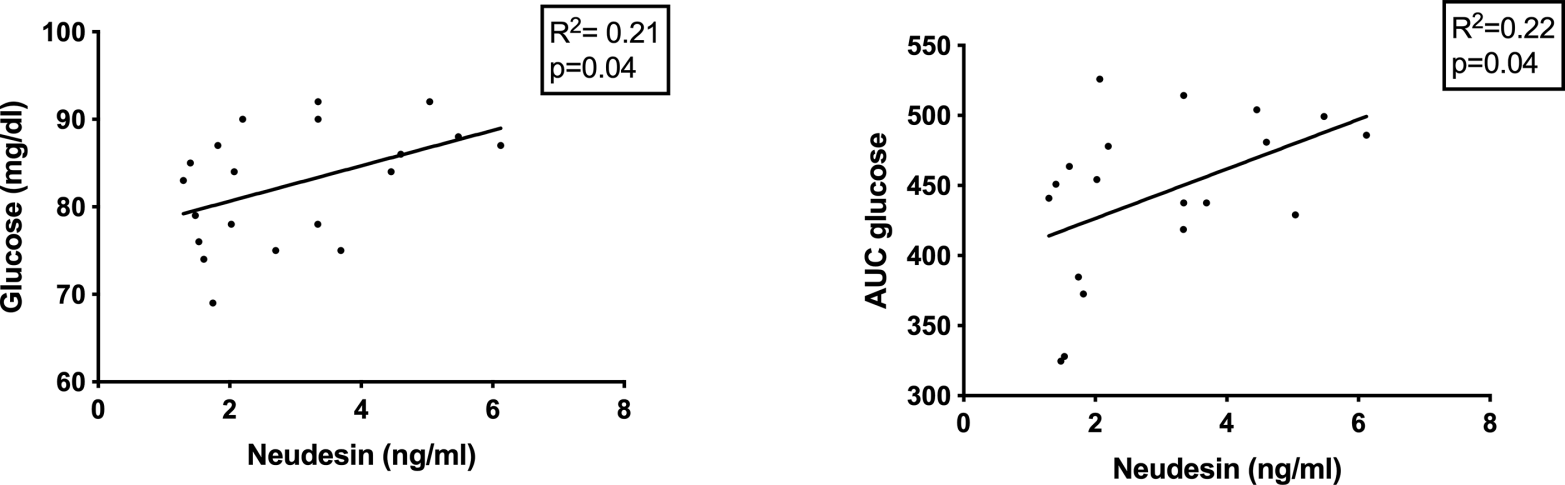
Correlations between plasma neudesin levels, glycemia and glucose AUC after oral load in obese/overweight children.

Interestingly, plasma neudesin levels in obese and overweight children were significantly directly correlated with basal glucose and glucose AUC after oral loading (**Figure 3**). No other significant correlations were found in either group.

The multivariate analysis in obese and overweight children group for neudesin according to BMI, glucose levels and HOMA-IR was performed. A statistically significant correlation between neudesin and glucose levels was confirmed (**Supplementary Table S1**).

## Discussion

While the assessment of circulating neudesin concentrations has already been performed in obese adolescents (21), to the best of our knowledge, this is the first evaluation of neudesin in obese children. This study shows that plasma levels of neudesin are significantly higher in obese and overweight children than in healthy children of normal weight (**Figure 2**).

Only a few studies have evaluated serum neudesin in the blood in humans, with discordant results. Kratochvilova et al. (2019) found no differences in serum neudesin levels among obese adults (81% with T2MD) undergoing endoscopic bariatric procedures, patients with functional hypoglycemia and healthy controls (17). On the other hand, recently Karatas et al. (20) reported higher serum levels of neudesin in adult patients with newly diagnosed T2MD compared to controls matched for age and BMI (20). However, the serum concentrations of neudesin detected by Karatas et al. (2021) are about thirty times higher than those reported in our paper and in most of the previously published papers (17, 19, 21–23). Celikkol et al. (21) reported significantly lower plasma levels of neudesin in a population of 58 obese adolescents compared to 30 normal weight adolescents (21). Nevertheless, this difference was attributable to the subgroup of 43 morbidly obese adolescents (BMI > 40 kg/m^2^), while the plasma levels of the remaining 15 obese adolescents were not significantly different from those of the control adolescents (21). As for children, the only study currently available was conducted on patients with T1DM, in which circulating levels of neudesin were found to be higher than in controls (22).

Kratochvilova et al. (17) further investigated the topic, evaluating the effects of chronic energy restriction (endoscopic bariatric intervention) and acute restriction (fasting test in hypoglycemic patients) on serum neudesin concentrations (17). The results were differential depending on the bariatric endoscopic procedure performed: after 6 months from endoscopic duodenal-jejunal bypass liner implantation, serum neudesin levels increased with a BMI decrease of approximately 5.2 kg/m^2^, even though no differences in neudesin gene expression in visceral and subcutaneous adipose tissue was detected before and after the implantation; conversely, at six months after gastric plication the serum levels of neudesin were not significantly different from the baseline ones and the mean BMI decreased by about 7.2 kg/m^2^. Interestingly, serum neudesin levels decreased significantly after 72-hour acute fasting in hypoglycemic patients. The authors therefore suggested a two-way behavior of neudesin in chronic versus acute energy restriction (17).

Experimental works carried out both *in vitro*, in pre-adipocyte cultures, and *in vivo*, in mice, have highlighted a possible link between neudesin, food intake and glucose metabolism (16, 32). However, it is not yet clear what the correlations between neudesin and IR are, due to the few works available in the literature, carried out with different experimental models. On the one hand, protection from IR induced by a high-fat diet and increased lipolysis and energy expenditure have been shown in neudesin gene knock-out mice. On the other hand, human data are quite conflicting. Kratochvilova et al. (17) found diverging correlations between neudesin and insulin serum levels in hypoglycemic patients undergoing acute fasting and in obese patients undergoing duodenal-jejunal bypass liner implantation: neudesin was indeed negatively correlated with insulin in the first group, while its levels varied according to insulin ones in the second group. Furthermore, neudesin mRNA levels in subcutaneous adipose tissue appeared to be inversely related to blood glucose in patients undergoing gastric plication. Serum concentrations of neudesin were negatively correlated with BMI and glycated hemoglobin in both obese patients with T2MD and children with T1MD (17, 22). In the latter group, no correlation was found between neudesin and glycemia, glycemic control or disease progression (22). Conversely, serum levels of neudesin showed a direct correlation with HOMA-IR and BMI (20). In partial agreement with the latter data, we found that plasma levels of neudesin in obese and overweight children are directly correlated with glycemia and glucose AUC after oral loading. Considering the available data, it can be stated that it is not yet possible to draw conclusions on the possible correlations between circulating neudesin and glucose metabolism.

It can be hypothesized that neudesin secretion into the bloodstream may represent an adaptation mechanism to overweight and IR. However, the controversial data published in the literature and the scarcity of large population studies do not allow to affirm with certainty the existence of a cause-effect relationship. It is therefore not yet clear whether higher plasma levels of neudesin in obese children are harmful or protective.

Future working hypotheses could include the identification of neudesin receptors and the search for mechanisms of neudesin-resistance, the instruction of longitudinal studies to understand the prognostic consequences of circulating neudesin levels, and the adoption more precise IR assessment techniques, such as hyperinsulinemic-euglycemic clamp.

Despite all precautions, our study, which is intended to be preliminary, may still be subject to certain biases and some potential restrictions should be considered. The number of subjects in the two groups is somewhat low, therefore the statistical power of the study is limited; this factor and the cross-sectional design of the study, do not allow to define a cause-effect relationship and our findings will need to be confirmed in a larger population, to better elucidate the role of neudesin in childhood metabolic disorders. Another possible bias could be the adoption of HOMA-IR and QUICKI, commonly used as indices of insulin resistance in large epidemiological studies, in a small population. Finally, our work was not designed to define the origin of circulating neudesin; therefore, further studies are needed to clarify how much of the plasma neudesin derives from synthesis in adipose tissue and how much comes from endocrine secretion from the central nervous system.

## Data Availability Statement

The raw data supporting the conclusions of this article will be made available by the authors, without undue reservation.

## Ethics Statement

The studies involving human participants were reviewed and approved by Dipartimento di Medicina e Chirurgia Traslazionale, Fondazione Policlinico Universitario A. Gemelli IRCCS, Institutional Review Board. Written informed consent to participate in this study was provided by the participants’ legal guardian/next of kin.

## Author Contributions

*Conceptualization*, AM, EV and CB; *methodology*, DC and AM; *software*, EV, DC and CB; *validation*, DC and AM; *formal analysis*, DC; *investigation*, EV, CB and CC; *resources*, EV, CB and CC; *data curation*, EV, DC and CB; *writing—original draft preparation*, EV and AM; *writing—review and editing*, EV, DC, CC and AM; *visualization*, EV and CB; *supervision*, DC and AM; *project administration*, DC, CC and AM; *funding acquisition*, DC and AM. All authors have read and agreed to the published version of the manuscript.

## Funding

Supported by Fondi di Ateneo Linea D1 to DC (N. R4124500530) and AM (N. RA124500855).

## Conflict of Interest

The authors declare that the research was conducted in the absence of any commercial or financial relationships that could be construed as a potential conflict of interest.

## Publisher’s Note

All claims expressed in this article are solely those of the authors and do not necessarily represent those of their affiliated organizations, or those of the publisher, the editors and the reviewers. Any product that may be evaluated in this article, or claim that may be made by its manufacturer, is not guaranteed or endorsed by the publisher.
