# Supplementary material for: Plasma Levels of Neudesin and Glucose Metabolism in Obese and Overweight Children
Source: Front Endocrinol (Lausanne). 2022 Jul 14;13:881524. doi: 10.3389/fendo.2022.881524 (PMC9331476; doi:10.3389/fendo.2022.881524)
Supplement: Supplementary file 1 [file Table_1.docx]

**SUPPLEMENTARY MATERIAL**

**Table S1**– Multivariate analysis for neudesin in obese/overweight group. SE = Standard Error, CI (95%) = 95% confidence interval.

|  | **Estimate** | **SE** | **CI (95%)** | **p value** |
| --- | --- | --- | --- | --- |
| **Blood Glucose (mg/dl)** | 0.108 | 0.946 | 0.008 to 0.207 | 0.03 |
| **BMI (kg/m^2^)** | 0.254 | 0.129 | -0.021 to 0.529 | 0.07 |
| **HOMA-ir** | 0.075 | 0.392 | -0.761 to 0.911 | 0.85 |
